# The Effects of Workplace Interventions on Low Back Pain in Workers: A Systematic Review and Meta-Analysis

**DOI:** 10.3390/ijerph182312614

**Published:** 2021-11-30

**Authors:** Fabrizio Russo, Giuseppe Francesco Papalia, Gianluca Vadalà, Luca Fontana, Sergio Iavicoli, Rocco Papalia, Vincenzo Denaro

**Affiliations:** 1 Department of Orthopaedic and Trauma Surgery, Campus Bio-Medico University of Rome, 00128 Rome, Italy; g.vadala@unicampus.it (G.V.); r.papalia@unicampus.it (R.P.); denaro@unicampus.it (V.D.); 2 Department of Public Health, Section of Occupational Medicine, University of Naples Federico II, 80121 Naples, Italy; Luca.fontana@unina.it; 3 Department of Occupational and Environmental Medicine, Epidemiology and Hygiene, Italian Workers’ Compensation Authority (INAIL), 00078 Rome, Italy; s.iavicoli@inail.it

**Keywords:** workplace interventions, low back pain, workers, work ability, systematic review, meta-analysis

## Abstract

This systematic review and meta-analysis aimed to analyze the effects of workplace interventions (WI) on clinical outcomes related to low back pain (LBP) in a worker population, and to assess socio-economic parameters as participants on sick leave, days of sick leave, and return to work following WI. A systematic literature search was performed to select randomized clinical trials that investigated the effectiveness of WI on return to work, sick leave, and working capacity of workers affected by nonspecific LBP. Fourteen articles were included in the review and meta-analysis. The meta-analysis showed improvements in pain (*p* = 0.004), disability (*p* = 0.0008), fear-avoidance for psychical activity (*p* = 0.004), and quality of life (*p* = 0.001 for physical scale and *p* = 0.03 for mental scale) for patients who underwent WI compared to controls. Moreover, the pain reduction following WI was statistically significant in the healthcare workers’ group (*p* = 0.005), but not in the other workers’ group. The participants on sick leave and the number of days of sick leave decreased in the WI group without statistical significance (*p* = 0.85 and *p* = 0.10, respectively). Finally, LBP recurrence was significantly reduced in the WI group (*p* = 0.006). WI led to a significant improvement of clinical outcomes in a workers’ population affected by LBP.

## 1. Introduction

Non-specific low back pain (LBP) is a common worldwide disorder with a significant impact on productivity, work ability, and quality of life [1]. Indeed, LBP is characterized by persisting pain, muscle weakness, reduction of physical activity [2], and sleep disorders, which have serious consequences on a person’s quality of life by limiting daily life and work activities [3]. The etiopathogenesis of nonspecific LBP is multifactorial including lifestyle risk factors (i.e., excess weight) [4] but also, according to the type of job, several occupational risk factors such as manual handling of heavy loads, awkward and prolonged postures (i.e., sustained sedentary work), whole-body mechanical vibrations, and work-related stress (i.e., psychosocial factors) [5]. The lifetime prevalence of LBP in the general population is high and was estimated at about 60–70% in industrialized countries [6]. Notably, in the literature, there is evidence that the prevalence of this disorder in particular working populations and/or industrial/productive fields such as construction, forestry or fishing, agriculture, and healthcare sectors is significantly higher than in the general population [7]. In particular, healthcare workers represent a job category easily susceptible to LBP biomechanical risk factors [8] with an annual prevalence of 40–50% [9], while the prevalence of LBP is estimated at about 34% in office workers [10]. LBP frequently causes sick leave and persistent or recurrent disability, representing an important socioeconomic burden [11].

Therefore, the prevention of work absenteeism due to LBP recurrence has become a public and occupational health priority worldwide [12]. The treatments for non-specific LBP usually consisted of non-surgical procedures, such as physical exercise, cognitive behavioral therapy, and pharmacological treatment [13,14,15]. Physical exercise at the workplace is considered an activity able to prevent occupational musculoskeletal disorders being able to enhance the physical capacity of workers. However, previous studies regarding occupational interventions showed contrasting results about the reduction of LBP symptoms following only physical exercise at the workplace [16,17,18]. This should not be surprising since, considering the numerous and different variables in workplaces that can play an important role in the onset of this disorder, it is likely that its prevention needs a multidisciplinary approach that exploits the simultaneous adoption of technical, organizational, procedural, and training measures. In this regard, several studies developed, applied, and evaluated this type of preventive strategies in different work environments consisting of workplace interventions (WI) that include workplace assessment, educational programs with ergonomic posture training sessions, physical activity at the workplace, and cognitive-behavioral therapy for the treatment of physical, psychological, occupational, and ergonomic risk factors. WI aim to prevent and/or manage LBP, reduce disabilities and fears for work and psychical activity, promote personalized action plans, and improve outcomes regarding work ability and quality of life.

Nevertheless, our current knowledge of the real effectiveness of WI remains rather fragmented and, at the same time, the understanding of the key factors or best combination of WI for achieving significant prevention or reduction of work-related LBP is equally limited. For this reason, in this context, we performed this systematic review and meta-analysis in order to critically evaluate the effects of WI on LBP in workers. In detail, the primary aim was to analyze the effects on clinical and occupational outcomes related to LBP in workers after the implementation of specific WI programs. The secondary endpoint was to assess the impact of WI on socio-economic parameters as participants on sick leave, days of sick leave, and return to work.

## 2. Materials and Methods

This study was carried out according to the Preferred Reporting Items for Systematic Reviews and Meta-Analysis (PRISMA) (Table S1) guidelines [19]. In this systematic review, we included randomized clinical trials (RCTs) that evaluated the effects of workplace interventions for workers suffering from LBP.

### 2.1. Inclusion Criteria 

The inclusion criteria were RCTs in the English language published in the last twenty years, which investigated the effectiveness of workplace interventions on return to work, sick leave, and the working capacity of workers affected by nonspecific LBP. The workplace interventions include technical interventions, physical exercise programs, behavioral training, educational programs, and participatory ergonomics. Exclusion criteria were represented by studies that enrolled patients with neurodegenerative diseases, previous spinal or brain surgery, or following spinal cord infections or injuries. We excluded studies that analyzed only physical or psychosocial activities as an intervention, and that only evaluated reducing sitting time as an outcome or the impact of a sit–stand workstation.

### 2.2. Search Methods 

A systematic literature search was executed using PubMed–Medline, Cochrane Central, Scopus, and Google Scholar. We used the following search strings: (“workplace” [MeSH Terms] or “workplace” [All Fields] or “workplaces” [All Fields] or “workplaces” [All Fields]) and (“interventions” [All Fields] or “interventions” [All Fields] or “interventive” [All Fields] or “methods” [MeSH Terms] or “methods” [All Fields] or “intervention” [All Fields] or “interventional” [All Fields]) and (“low back pain” [MeSH Terms] or (“low” [All Fields] and “back” [All Fields] and “pain” [All Fields]) or “low back pain” [All Fields]). The reference lists of the included RCTs were detected to obtain further eligible studies. After removing duplicates, two independent investigators reviewers (G.P. and F.R.) checked the abstracts of potentially included studies. Any divergence was discussed with the third review author (G.V.). Finally, two review authors (G.P. and F.R.) read the full articles in order to select the included studies for this review and meta-analysis. 

### 2.3. Data Collection, Analysis, and Outcomes 

Two independent reviewers (G.P. and F.R.) conducted data extraction. The following data were extracted from the included studies: Authors, year of publication, type of study, level of evidence, numbers of participants in study and control groups, age and sex of participants, types of workers, intervention in the experimental and in the control group, follow-up, and results. LBP, disability, fear-avoidance beliefs, quality of life, and work ability were assessed as outcomes in the included studies. Finally, participants on sick leave, days of sick leave, LBP recurrence, and return to work were compared between workplace intervention and control groups.

### 2.4. Risk of Bias Assessment 

The risk of bias of the included RCTs was evaluated by two authors (G.P. and F.R.) by the guideline for systematic reviews in the Cochrane Back and Neck Group [20]. This tool assesses the following types of biases: selection bias, performance bias, attrition bias, detection bias, and reporting bias. The trials were judged at low, unclear, or high risk of bias in relation to the risk of bias of the various domains.

### 2.5. Statistical Analysis

A meta-analysis was performed using the Review Manager (RevMan) software Version 5.4.1 (The Nordic Cochrane Center, The Cochrane Collaboration, 2014, Copenhagen, Denmark). Low back pain, disability, fear-avoidance beliefs, quality of life, and work ability between the experimental and the control groups were calculated as continuous outcomes. Instead, participants on sick leave, days of sick leave, LBP recurrence, and return to work were evaluated as dichotomous outcomes. The continuous data are presented as the standard mean difference (SMD) with 95% confidence intervals due to the adoption of diverse scores in the included studies. The outcomes expressed with negative mean values of SMD present a higher improvement with lower values. Dichotomous data are shown as odds ratio (OR) with 95% confidence intervals. For the calculation of the weight of the samples of the trials, for the days of sick leave, we used mean days of sick leave per participant as events and the total number of days of follow-up per participant as the total. A subgroup analysis was performed to evaluate LBP in healthcare workers and other kinds of workers. The heterogeneity was calculated using the I^2^ test. A fixed-effect model was adopted for low heterogeneity (I^2^ < 55%); otherwise, a random-effect model was involved. The statistical significance of the results was set at *p* < 0.05. 

### 2.6. Quality Assessment

The quality of the evidence and strength of recommendation of the selected outcomes were analyzed by the GRADE (Grading of Recommendations Assessment, Development, and Evaluation) assessment. Five elements (risk of bias, inconsistency, indirectness, imprecision, and publication bias) were assessed for each result and were categorized as not serious, serious, or very serious. The outcomes for RCTs received an initial ranking of high quality of evidence, which could be downgraded to moderate, low, or very low concerning the valuation of the five items.

## 3. Results

### 3.1. Results of the Search 

**Figure 1:**
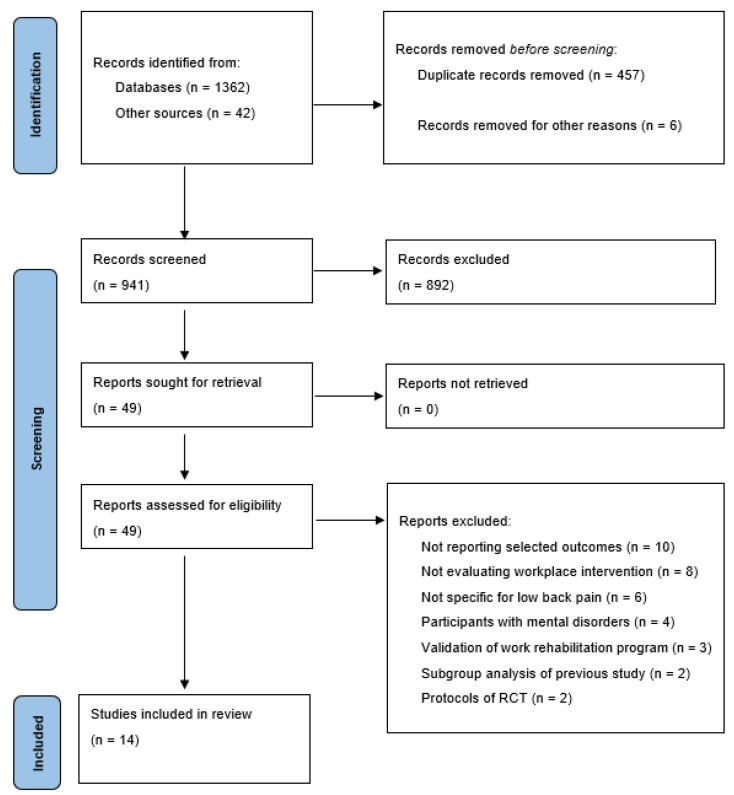
Preferred Reporting Items for Systematic Review and Meta-Analysis (PRISMA) flow diagram.

The literature search generated 691 articles. After removing duplicates, the reviewers screened titles and abstracts of 673 papers, and chose 41 eligible articles that were read in full. Afterwards, 27 studies were excluded for the following reasons: Not reporting selected outcomes (*n* = 9), not evaluating workplace interventions (*n* = 6), not specific for LBP (*n* = 4), patients with mental disorders (*n* = 3), validation of work rehabilitation program (*n* = 2), subgroup analysis of previous study (*n* = 2), and protocols of RCT (*n* = 2). Finally, 14 articles were included in the review and meta-analysis (Figure 1). 

### 3.2. Demographic Data

The total number of participants in all the studies was 3197, divided into 1837 in the study group and 1360 in the control group (Table 1). Patients’ ages ranged from 29.6 to 52 years in the study groups, and from 26.6 to 51 in the control groups. The percentages of men in the studies ranged from 99% to 0% in the intervention groups and from 98.4% to 0% in the control groups. Therefore, important heterogeneity in the gender of the participants of the included studies was reported. The workers analyzed were distributed as follows: Nursing assistants or healthcare workers in six studies (43%), office workers in two studies (14%), employees in the automotive industry in one study (7%), workers at a manufacturing company in one study (7%), physically demanding workers in one study (7%), and workers (without specification of the type of job) in three studies (22%).

### 3.3. Workplace Intervention Program

Workplace intervention protocols including multidisciplinary interventions consisted of a combination of the following programs: Work-related evaluation and a workplace assessment with work modifications (four studies); an educational program and ergonomic posture training sessions (six studies); a supervised intervention of exercise sessions of muscle strengthening, flexibility, segmental stabilization, and endurance training on the workplace (six studies); and behavioral counseling and cognitive-behavioral therapy for LBP or stress self-management (two studies). The mean follow-up was 11.3 months and ranged from 3 to 24 months. 

### 3.4. Clinical Outcome Data

Clinical outcomes were diversified in the included studies. LBP was assessed in 10 studies, using the Visual Analogue scale (VAS) [21,22,23,24,25], Numeric Rating Scale (NRS) [26,27], Multidimensional Pain Inventory (MPI-D) [28], and Cornell musculoskeletal discomfort questionnaire [29]. Disability was evaluated in five trials, by the Quebec Disability Scale [23,24,27] and Oswestry Disability Index (ODI) [25,30]. The work subscale of Fear-Avoidance Beliefs Questionnaire (FABQ-W) was assessed in four studies [22,24,26,28], while the physical activity subscale (FABQ-P) was assessed in three studies [24,26,28]. Work ability was reported in two studies, using the Work Ability Index (WAI) [22] and subjective working capacity [30]. Quality of life was assessed in three studies [24,26,28] using physical and mental scales of the Short-Form-36 (SF-36) or Short-Form-12 (SF-12) Health Survey. 

### 3.5. Methodological Evaluation

Through the guideline for systematic reviews in the Cochrane Back and Neck Group, four studies (28.6%) were at low risk of bias (A), eight studies (57.1%) were judged at unclear risk of bias (B), and only two studies (14.3%) were at a high risk of bias (C) (Table 2). Precisely, random sequence generation was adequate in all the studies (100%). Allocation concealment was graded as adequate in all the studies except one (93%). Blinding for patients and care providers was not adequate in all studies, due to the modality of the interventions. Blinding for outcome assessment resulted in being adequate in all the studies (100%). Selective reporting was evaluated as adequate in six studies (43%). Other sources of bias were adequate in four trials (28.6%).

**Table 1:** Main characteristics and clinical results of the included studies.

Characteristics of Working Population		WI		Follow-Up	Results	References
Study Group	Control Group	Study Group	Control Group			
37 (29.7% M, 70.3% F) nursing staff working in the operating room (age 31.45 ± 8.19)	37 (18.9% M, 81.1% F) nursing staff working in the operating room (age 26.64 ± 5.83)	Ergonomics educational program	No intervention	3 months	The IG reported a reduction in the prevalence of musculoskeletal disorders, in particular of LBP (*p* = 0.000).	Abdollahi et al. (2020) [31]
96 (53% M, 47% F) unspecified workers (age 44 ± 8.6)	100 (33% M, 67% F) unspecified workers (age 41.2 ± 10.7)	Workplace intervention: workplace assessment, work modifications, and case management	Usual care	12 months	Time until return to work for workers with WI was 77 versus 104 days for workers without this intervention (*p* = 0.02). Functional status and pain intensity improved more in workers who received a WI, than in workers without this intervention.	Anema et al. (2007) [21]
13 (15% M, 85% F) office workers (age 52 ± 9)	14 (29% M, 71% F) office workers (age 51 ± 13)	Behavioural counselling, sit-stand desk attachment and cognitive behavioral therapy for LBP self-management	No intervention	6 months	The relative decrease in ODI from baseline was 50% in the IG and 14% in the CG (*p* = 0.042). LBP was not significantly reduced in IG versus CG, though small-to-moderate effect sizes favoring the IG were observed.	Barone Gibbs et al. (2018) [16]
171 (23% M, 77% F) healthcare workers from hospitals (age 47.1 ± 8.5)	171 (22% M, 78% F) healthcare workers from hospitals (age 47.3 ± 8.5)	Exercise training sessions in the workplace, and a home-based self-managed EP	Usual care	24 months	35 workers in the IG and 31 workers in the CG had at least one LBP recurrence with sick leave. The intervention was effective in reducing fear avoidance with a mean reduction of −3.6 points in the IG compared with −1.3 points in the CG (*p* < 0.05).	Chaléat-Valaye et al. (2016) [15]
28 (50% M, 50% F) employed patients (age 41.46 ± 11.93)	23 (43.5% M, 56.5% F) employed patients (age 48.30 ± 10.14)	Individually targeted vocational sessions in conjunction with group rehabilitation for LBP	Group Rehabilitation	6 months	The IG had a better outcome for disability or pain and fear-avoidance	Coole et al. (2012) [13]
92 (8.7% M, 91.3%F) nurses (age 37.9 ± 11.6)	91 (6.6% M, 93.4%F) nurses (age 41.1 ± 10.8)	Psychological units, segmental stabilization exercises units, and ergonomic and workplace-specific units (plus General Physical EP)	General Physical EP	12 months	For the primary study end point of pain interference, the effect size at 12 months after intervention was 0.58 in the MP and 0.47 in the EP.	Ewert et al. (2009) [19]
153 (68% M, 32% F) physically demanding workers (age 45.3 ± 10.1)	152 (67.1% M, 32.9% F) physically demanding workers (age 45.7 ± 10.5)	Occupational medicine consultations, a work-related evaluation and workplace intervention plan, an optional workplace visit, and a physical activity program	No intervention	6 months	Both groups showed improvements in average pain score, disability, fear-avoidance beliefs for physical activities and work; no statistically significant difference was found between the groups.	Hansen et al. (2019) [17]
59 (100% F) healthcare and social care professionals at healthcare centers (age 46 ± 7.9)	61 (100% F) healthcare and social care professionals at healthcare centers (age 46.5 ± 7)	Physical training, relaxation training, and cognitive-behavioral stress management methods	Physical exercise and passive treatment	24 months	In the MR group, statistically significant differences (at least *p* < 0.05) were found during the follow-up in ODI, subjective working ability and beliefs in future working ability.	Kaapa et al. (2006) [21]
301 (99% M, 1% F) manufacturing company workers (age 35.4)	315 (98.4% M, 1.6% F) manufacturing company workers (age 36.5)	Training sessions of participatory workplace improvement-based provision of ergonomic training and ergonomic action checklists on workplace improvement activities	Usual care	12 months	In the IG the incident rate ratio of participatory workplace improvements for the LBP category was significantly elevated after the training sessions, but decreased during the 10-month follow-up period.	Kajiki et al. (2017) [23]
107 (42% M, 58% F) employees at primary health care centers (age 44)	57 (40% M, 60% F) employees at primary health care centers (age 43)	Exercises for improving the function of the deep abdominal muscles and establishing symmetric use of the back (plus a worksite visit)	Usual care	24 months	There were no differences between the three treatment arms regarding the intensity of pain and the perceived disability. The average number of days on sick leave was lower in the IGs than in the CG (*p* = 0.03).	Karjalainen et al. (2004) [24]
37 (70.3% M, 29.7% F) employees working in assembly positions in the automotive industry (age 45.1 ± 9.11)	38 (44.8% M, 55.2% F) employees working in assembly positions in the automotive industry (age 45.34 ± 8.80)	Supervised WI of muscle strengthening, flexibility, and endurance training	No intervention	6 months	Significant beneficial effect (*p* < 0.025) for the IG at 2 and 6 months in pain parameters, specific flexibility, and in back functions.	Nassif et al. (2011) [18]
646 (gender not available) employees in two municipalities (age not available)	211 (gender not available) employees in two municipalities (age not available)	Educational meetings, peer support and access to an outpatient clinic	Usual care	12 months	The IG had significantly fewer days of sick leave at the three-month (4.9 days, *p* = 0.001) and six-month (4.4 days, *p* = 0.016) follow-ups compared with the CG.	Ree et al. (2016) [25]
34 (gender not available) white collars (age 29.64 ± 0.90)	28 (gender not available) white collars (age 28.74 ± 0.82)	Office-based stretching exercises mechanisms to rise the range and flexibility of motion in the muscles of the back plus “total workplace Occupational Safety and Health and ergonomic intervention”	No intervention	6 months	Significant differences were seen in pain scores for lower back (MD −6.87; 95% CI −10 to −3.74) between the combined exercise and ergonomic modification and CGs.	Shariat et al. (2017) [20]
63 (82.5% M, 17.5% F) nursing assistants (age not available)	62 (75.8% M, 24.2% F) nursing assistants (age not available)	Multidisciplinary intervention consisted of an educational program and ergonomic posture training	Usual care	6 months	The comparison tests showed significant change from baseline in reduction of work-related LBP intensity following the multidisciplinary program, with scores of 5.01 ± 1.97 to 3.42 ± 2.53 after 6 months on the visual analog scale in the IG (*p* < 0.001) and no significant change in CGs.	Shojaei et al. (2017) [14]

All the studies reported in the table were randomized clinical trials and have a level of evidence = I; F: Female; M: Male; IG: Intervention Group; LBP: Low Back Pain; WI: Workplace Intervention; EP: Exercise Program; CG: Control Group; MP: Multimodal Program; ODI: Oswestry Disability Index; MR: Multidisciplinary Rehabilitation.

### 3.6. Effect of Intervention

**Figure 2:**
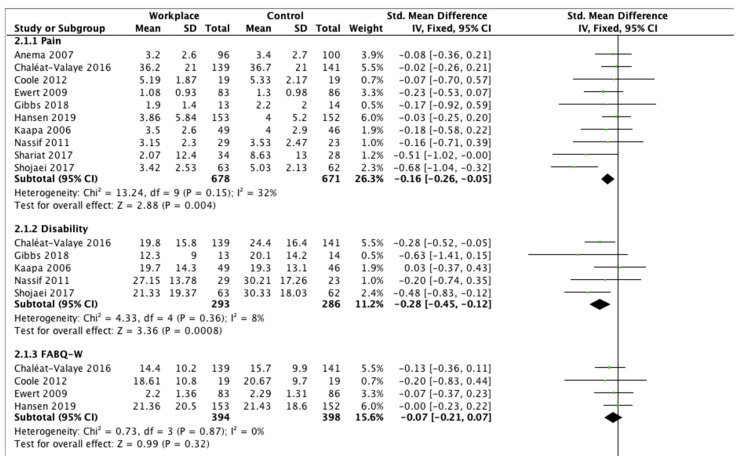

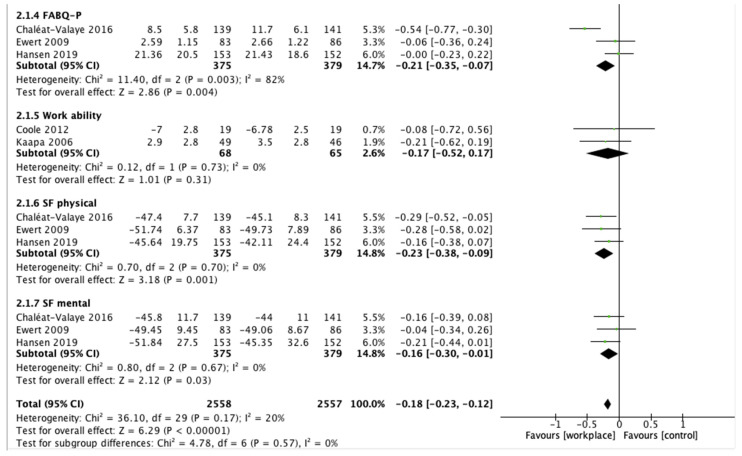
Outcome measurements.

The meta-analysis demonstrated the effectiveness of workplace interventions on LBP, disability, the Fear-Avoidance Beliefs Questionnaire for work and psychical activity, work ability, and quality of life compared to controls (Figure 2). Pain decreased significantly in the intervention group in comparison with the control group (SMD −0.16, 95% CI −0.26 to −0.05, *p* = 0.004). Disability scores showed significant improvements for workplace interventions compared to controls (SMD −0.28, 95% CI −0.45 to −0.12, *p* = 0.0008). FABQ-W demonstrated lower fear-avoidance beliefs about work in workers who underwent WI compared to controls (SMD −0.07, 95% CI −0.21 to 0.07), but no significant differences (*p* = 0.32). FABQ-P showed a significant reduction of fear-avoidance beliefs about physical activity in the experimental group (SMD −0.21, 95% CI −0.35 to −0.07, *p* = 0.004). Work ability presented improvements in favor of the intervention group (SMD −0.17, 95% CI −0.52 to 0.17), without significant differences (*p* = 0.31). Short-Form Health Survey results showed statistically significant improvements in quality of life for both the scales for the participants in the workplace intervention group (SMD −0.23, 95% CI −0.38 to −0.09, *p* = 0.001 for physical scale, and SMD −0.16, 95% CI −0.30 to −0.01, *p* = 0.03 for mental scale, respectively). Finally, evaluating the clinical outcomes in totality, a significant difference was reported in favor of the workplace intervention group compared to the controls (*p* < 0.00001).

**Figure 3:**
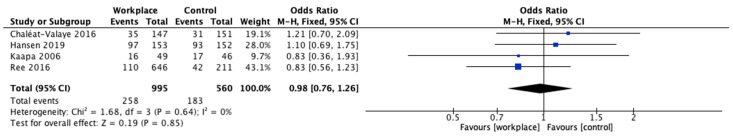
Participants on sick leave.

**Figure 4:**
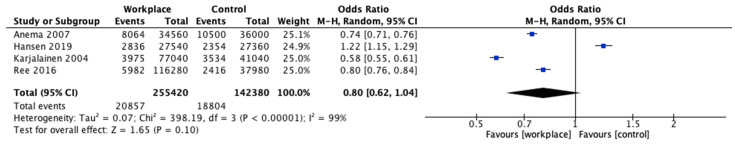
Days of sick leave.

**Figure 5:**
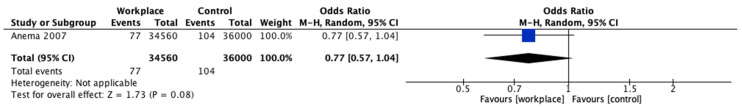
Return to work.

**Figure 6:**
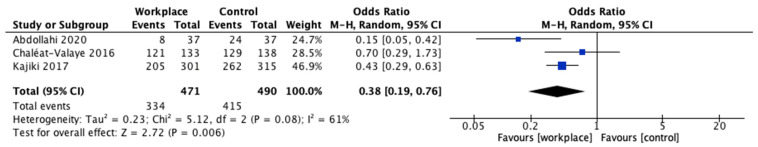
Low back pain recurrence.

The analysis of the participants on sick leave showed a reduction for patients who underwent intervention programs (OR 0.98, 95% CI 0.76 to 1.26) but no significant differences (*p* = 0.85) (Figure 3). The number of total days of sick leave decreased in the WI group (OR 0.80, 95% CI 0.62 to 1.04) without statistical significance (*p* = 0.10) (Figure 4). Return to work was analyzed in only one study, which reported a better result in favor of the study group (OR 0.77, 95% CI 0.57 to 1.04, *p* = 0.08) (Figure 5). Finally, LBP recurrence was significantly reduced in the intervention group compared to controls (OR 0.38, 95% CI 0.19 to 0.76, *p* = 0.006) (Figure 6).

**Figure 7:**
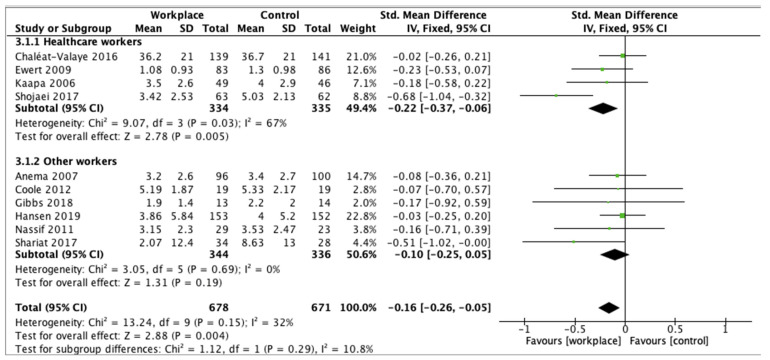
Subgroup analysis for low back pain.

In conclusion, the test for subgroup differences showed no statistically significant subgroup effect for LBP (*p* = 0.29). However, the pain reduction after WI was statistically significant in the healthcare workers’ group (*p* = 0.005), but no difference was reported in the other workers’ group (Figure 7).

### 3.7. Quality Assessment

GRADE was applied to evaluate the quality of the evidence given in the included RCTs (Table 3). It produced seven comparisons for continuous data and three for dichotomous data. Regarding clinical outcomes, disability, SF physical, and SF mental maintained a high quality of evidence, while pain obtained high-quality evidence because it received an upgrade due to the large effect. FABQ-W and FABQ-P were downgraded by one level for risk of bias and inconsistency, thus reporting a moderate quality of evidence. Finally, work ability presented a low quality of evidence, due to risk of bias and imprecision. In contrast, the outcomes of participants on sick leave and days of sick leave achieved low quality of evidence, while LBP recurrence reported a very low quality of evidence.

## 4. Discussion

The primary aim of this systematic review and meta-analysis was to assess the effects of WI on workers in terms of clinical outcomes. The secondary endpoint was the interpretation of socio-economic parameters as participants on sick leave, days of sick leave, LBP recurrence, and return to work following specific workplace programs. Studies that analyzed only physical or psychosocial activities at the workplace or evaluated the effectiveness of a sit–stand workstation were not included since WI must be analyzed in its entirety in order to provide the best support to workers and obtain the best benefits in terms of LBP, work ability, and return to work.

Employees who underwent WI experienced improvements in LBP, disability, fear-avoidance beliefs, quality of life, and work ability compared to controls, with a significant increase for all the reported scores. The meta-analysis proved that the scores regarding LBP, disability, FABQ-P, and SF physical and mental obtained the most statistical significance compared to controls, showing the best improvements after WI. On the other hand, FABQ-W and work ability outcomes did not show significant differences compared to control groups. Therefore, it has been shown that WI led to excellent results in symptom reduction, daily living activities, and quality of life, but it remains a subjective limitation for workers to perform their job activities. Moreover, the meta-analysis showed non-significant improvements in participants on sick leave, days of sick leave, and return to work after WI. This could further demonstrate that workers did not feel able and ready to undergo workloads, although they have experienced a significant reduction in LBP and a global increase in quality of life. The subgroup analysis for LBP, even if in absence of a significant subgroup effect, showed a greater reduction of pain after WI in healthcare workers compared to other workers. Therefore, workplace interventions seem to ensure greater benefits for a population of nurses and healthcare workers, but further and more specific trials are needed to demonstrate these results. However, for the other clinical outcomes, it was not possible to observe a difference between the different kinds of works.

Furthermore, all the clinical outcomes showed high (pain, disability, SF physical, and SF mental) or moderate (FABQ-W and FABQ-P) quality of evidence and strength of recommendation at GRADE—except for work ability, which had a low quality of evidence—justifying a recommendation of workplace interventions in workers with LBP. On the other hand, GRADE reported a low quality of evidence for participants on sick leave and days of sick leave and very low quality for LBP recurrence. Finally, it should be noted that by the guideline for systematic reviews in the Cochrane Back and Neck Group, only two studies have been judged at a high risk of bias, showing an acceptable overall quality of the included studies. Almost all the studies showed a low risk of bias for random sequence generation and allocation concealment. However, in all studies, the risk of bias was high for blinding for patients and care providers, due to the impossibility to blind patients to the interventions that they were receiving. Instead, in all the trials, the outcomes were reported by assessors blinded to the group allocation.

In their review, Gobbo et al. [16] showed that exercise programs in the workplace reduce LBP symptoms, improve muscle strength and flexibility, and increase the quality of life in office workers. Contrarily, the meta-analysis performed by Maciel et al. [17] showed that physical exercise at the workplace did not reduce the occurrence of LBP (*p* < 0.4). Sowah et al. [18] evaluated occupational interventions as treatments for the prevention of LBP and demonstrated that exercise interventions, with or without educational interventions in the workplace, have the potential to prevent LBP. More specifically, Roman-Liu et al. [32] proved strong differences in effects among intervention strategies. In fact, they showed that technical modifications of the workstand and education based on practical training represent more effective strategies for LBP prevention than behavioral and physical training. Finally, in a meta-analysis conducted by Parry et al. [33], they did not show evidence that interventions to increase standing or walking in the workplace reduce musculoskeletal symptoms in sedentary workers. 

Certain limitations may hinder the interpretation of data. The limitations of this study are related to the heterogeneity in the population of workers of the included RCTs. Indeed, three studies [21,22,25] did not specify the job of the participants, six studies involved nursing assistants or healthcare workers [23,24,28,30,31,34], while the patients in the remaining five trials [26,27,29,35,36] practiced other kinds of jobs. Other heterogeneities concerned the age and sex of the participants. In fact, the mean age, ranging from 26.6 to 52 years, correlated with different grades of LBP, which may need diverse treatments. Moreover, two studies showed a clear predominance of women [28,30], one study enrolled almost all men [35], and two studies did not report the gender distribution of the participants [29,36]. Due to the multiple works analyzed and the differences in the population groups, the workplace interventions performed in the various trials were very miscellaneous and not homogeneous. Moreover, two studies showed a clear predominance of women [26,28], one study enrolled almost all men [33] and two studies did not report the gender distribution of the participants [27,34]. Due to the multiple works analyzed and to the differences in the population groups, the follow-up was one year or less in 11 studies (78.5%), not allowing the comprehension of long-term effects of WI on LBP, disability, quality of life, and work ability. 

The evaluation of findings provided by the studies included in this review clearly showed that different types of WI determine a beneficial effect both on clinical outcomes and socio-economic parameters related to LBP in workers. However, in consideration of the considerable heterogeneity (in terms of working population, socio-demographic characteristics, and diversification of WI) of the studies, trying to establish which is the best approach in terms of effectiveness in preventing LBP (and therefore in reducing its multiple negative effects) is a rather challenging task that can also lead to drawing conclusions that are not entirely correct. Indeed, in this regard, it should be taken into account that the degree of effectiveness of the different WI strategies may be affected by numerous factors such as the socio-demographic characteristics of the working population (e.g., gender, age, education level, presence of chronic degenerative diseases), the working activities carried out by employees, which determine a greater or lesser exposure to occupational risk factors for LBP (e.g., manual handling of heavy loads, awkward and prolonged postures, whole-body mechanical vibrations, work-related stress), and the number and type of WI (e.g., technical interventions, procedural measures, organizational tools, educational programs) [5]. Consequently, even if the literature data suggest, for example, that an engineering redesign of workstations is more effective than participatory ergonomics or that a tailored physical exercise achieves better results when coupled with cognitive and behavioral training or even that strength exercise is more beneficial than cardiorespiratory exercise, it is not obvious that a WI strategy based on the aforementioned indications will achieve the same level of effectiveness in all workplaces or working populations. Therefore, in our opinion, a prevention program based on WI to be truly decisive in reducing the negative effects of LBP in workers cannot be limited to replicating the same intervention strategy in all workplaces. In this regard, we believe that the design of an adequate WI approach must be based on a flexible decision-making process, which, starting from the occupational risk assessment and taking into account the characteristics of the working population, identifies, on the basis of the evidence of the literature, the best possible combination of the use of the different WI. Indeed, WI should be targeted for a specific work, with the simultaneous and combined presence of all the programs, such as a technical intervention, physical exercise, behavioral training, and educational and participatory ergonomics, in order to treat and prevent the LBP in the totality of its manifestations at workplace. 

## 5. Conclusions

This systematic review demonstrated that workplace interventions led to a significant improvement of clinical outcomes in a worker population affected by LBP. The meta-analysis showed strong evidence that WI improved LBP, disability, and quality of life in workers. However, a statistical increase in purely working parameters has not been described, testifying to the fact that despite the pain decreased, workers were still afraid to fully return to work. WI should be practiced in order to prevent and treat musculoskeletal symptoms, which could reduce the work ability and increase the number of sick leave days for the workers. However, workplace interventions standardized for specific works are needed, and the follow-up should be longer to evaluate the long-term effects of WI on clinical and working outcomes. 

## Figures and Tables

**Table 2:** Guideline for systematic reviews in the Cochrane back and neck group.

Study	Randomization	Allocation	Patient Blinded	Care Provider Blinded	Outcome Assessor Blinded	Drop-Out Rate	All Randomized Participants Analyzed in the Group	Free of Selective Reporting	Groups Similar at Baseline	Cointerventions Avoided	Compliance	Timing of Outcome Assessment	Other Sources of Bias	Risk of Bias
Abdollahi	Y	Y	N	N	Y	Y	Y	U	Y	Y	Y	Y	U	B
Anema	Y	Y	N	N	Y	Y	Y	U	Y	Y	N	Y	U	B
Barone Gibbs	Y	Y	N	N	Y	Y	Y	Y	Y	Y	Y	Y	U	A
Chaléat-Valaye	Y	Y	N	N	Y	U	Y	Y	Y	Y	Y	Y	Y	A
Coole	Y	U	N	N	Y	N	Y	U	Y	Y	N	Y	U	C
Ewert	Y	Y	N	N	Y	U	Y	Y	Y	Y	Y	Y	U	B
Hansen	Y	Y	N	N	Y	Y	Y	Y	Y	Y	Y	Y	Y	A
Kaapa	Y	Y	N	N	Y	U	Y	Y	Y	Y	Y	Y	U	B
Kajiki	Y	Y	N	N	Y	Y	Y	U	Y	Y	Y	Y	Y	A
Karjalainen	Y	Y	N	N	Y	Y	Y	U	Y	Y	Y	Y	U	B
Nassif	Y	Y	N	N	Y	N	Y	Y	Y	Y	U	Y	U	B
Ree	Y	Y	N	N	Y	U	Y	U	Y	Y	U	Y	U	C
Shariat	Y	Y	N	N	Y	N	Y	U	Y	Y	U	Y	Y	B
Shojaei	Y	Y	N	N	Y	Y	Y	U	Y	Y	Y	Y	U	B

Y: Yes; N: No; U: Unsure.

**Table 3:** GRADE.

Outcomes	N. of Participants (Studies)	Risk of Bias	Inconsistency	Indirectness	Imprecision	Other Considerations	Quality
Pain	1349 (10 RCT)	serious	not serious	not serious	not serious	not serious	⨁⨁⨁⨁ high *
Disability	579 (5 RCT)	not serious	not serious	not serious	not serious	not serious	⨁⨁⨁⨁ high
FABQ-W	792 (4 RCT)	serious	not serious	not serious	not serious	not serious	⨁⨁⨁◯ moderate
FABQ-P	754 (3 RCT)	not serious	serious	not serious	not serious	not serious	⨁⨁⨁◯ moderate
Work ability	133 (2 RCT)	serious	not serious	not serious	serious	not serious	⨁⨁◯◯ low
SF physical	754 (3 RCT)	not serious	not serious	not serious	not serious	not serious	⨁⨁⨁⨁ high
SF mental	754 (3 RCT)	not serious	not serious	not serious	not serious	not serious	⨁⨁⨁⨁ high
Participants on sick leave	1555 (4 RCT)	serious	not serious	not serious	serious	not serious	⨁⨁◯◯ low
Days of sick leave	1526 (4 RCT)	serious	serious	not serious	not serious	not serious	⨁⨁◯◯ low
LBP recurrence	961 (3 RCT)	serious	serious	not serious	serious	not serious	⨁◯◯◯ very low

N.: Number; FABQ-W: Fear-Avoidance Beliefs Questionnaire Work subscale; FABQ-P: Fear-Avoidance Beliefs Questionnaire Physical activity subscale; SF: Short Form; LBP: Low Back Pain; RCT: Randomized clinical trial. * Upgrade due to large effect.

## Supplementary Materials

The following are available online at https://www.mdpi.com/article/10.3390/ijerph182312614/s1, Table S1: PRISMA Checklist.
